# Tidings from the Tides–*De novo* transcriptome assembly of the endemic estuarine bivalve *Villorita cyprinoides*

**DOI:** 10.1038/s41597-024-03541-4

**Published:** 2024-07-02

**Authors:** Summaya Rahuman, Jeena N. S., Wilson Sebastian, Eldho Varghese, Asokan P. K.

**Affiliations:** 1 Indian Council of Agricultural Research – Central Marine Fisheries Research Institute, Kochi, 682 018 Kerala India; 2 Mangalore University, Mangalagangotri, Mangalore, 574 199 Karnataka India; 3 Centre for Marine Living Resources and Ecology, Kochi, 682508 Kerala India

**Keywords:** Transcriptomics, RNA sequencing

## Abstract

The Indian black clam *Villorita cyprinoides* Gray, 1825, is an economically valuable estuarine bivalve that faces challenges from multiple stressors and anthropogenic pressures. However, limited genomic resources have hindered molecular investigations into the impact of these stressors on clam populations. Here, we have generated the first transcriptomic reference datasets for *V. cyprinoides* to address this knowledge gap. A total of 25,040,592 and 22,486,217 million Illumina paired-end reads generated from two individuals were assembled using Trinity and rnaSPAdes. From the 47,607 transcripts identified as Coding Domain Sequences, 37,487 returned positive BLAST hits against six different databases. Additionally, a total of 14,063 Single Sequence Repeats were identified using GMATA. This study significantly enhances the genetic understanding of *V. cyprinoides*, a potential candidate for aquaculture that supports the livelihoods of many people dependent on small-scale fisheries. The data generated provides insights into broader genealogical connections within the family Cyrenidae through comparative transcriptomics. Furthermore, this transcriptional profile serves as baseline data for future studies in toxicological and conservation genetics.

## Background & Summary

Estuarine regions are ecotone zones with rich biodiversity that possess great ecological and economic values^1^. Among the wider range of species communities within an estuarine system, the malacofauna represents a prominent fisheries resource, rendering a significant contribution to Small Scale Fisheries (SSF)^2–5^. Besides, these sentinel soft-bottom bivalve communities provide various ecosystem services including biomonitoring of pollution and assessment of toxin accumulation while improving water quality through filtration^6,7^. Being a biodiversity-rich ecosystem, the detrimental effects of climatic changes and anthropogenic pressures exerted on estuarine communities, particularly in bivalves, are implausible^8,9^. The aforementioned factors have a pronounced effect on endemic as well as economically exploited species, which brings us to the species of interest of this work- *Villorita cyprinoides* Gray, 1825.

Popularly known as the Indian black clam, *Villorita cyprinoides* is an endemic cyrenid clam inhabiting the estuaries of Peninsular India. Being a readily available and affordable protein source, this artisanal fishery accounts for more than 70 percent of the Indian clam fishery, making it economically valuable and overutilized^10^. However, the wild population of the clam faces significant challenges including multiple climatic stressors and anthropogenic pressures such as overfishing and habitat destruction. This inland fishery has been overexploited and reported with rapid and fragmentary population decline despite implementing a minimum legal catch size of 10 mm as a preventive measure^11,12^. Niche fragmentation, climate change, and associated environmental stressors have drastically altered larval development and threatened the existence of this organism^13^. To rejuvenate black clam resources in fishery areas, immediate actions such as re-laying have been undertaken, and initial breeding standardization studies are in progress^14^. However, due to limited genomic resources, it is difficult to ensure the conservation and sustainability of *Villorita* given the current significant risk in its population.

Apart from the evident economic importance, *Villorita* is also considered an excellent sentinel organism, monitoring ecotoxicological changes and ensuring ecosystem health^15,16^. Recent studies have discovered elevated levels of metals in black clams, leading to concerns about the overall health of the ecosystem^17^. Moreover, numerous earlier investigations have emphasized the substantial buildup and susceptibility of black clams to biologically essential metals like zinc (Zn) and copper (Cu)^18,19^. Though they are reported to exhibit resilience to environmental perturbations, various climatic stressors are directly influencing their survival^11^. To address these issues, molecular responses, fluctuations in the internal environment, signaling pathways, and associated genes need to be monitored when clams are probed for pollutants and environmental stressors^20^. This requires the elucidation of robust Reference Transcriptomic Datasets (RTD) that provide a basic notion of transcripts produced by a non-model organism^21^. In the past decades, high throughput next-generation sequencing (NGS) technologies have enabled large-scale sequencing of genomic/transcriptomic data with efficiency and low cost^22^, making the “omics” data more accessible^23^, and facilitating genetic level investigation easier. However, the transcriptome profile of brackish water clams has received less attention^24,25^.

In this study, we aim to characterize the first comprehensive transcriptome sets of the commercially important bivalve, *V. cyprinoides* endemic to peninsular India. A total of 25,040,592 and 22,486,217 million raw paired-end reads were generated from two *V. cyprinoides* samples using Illumina short-read sequencing, respectively. These reads were then assembled using the *de novo* transcript reconstruction method. Despite the lack of a reference genome, the transcriptomic data obtained in this study will serve as an important genomic resource and act as a prerequisite for further ecotoxicological, gene expression, and population studies on this species.

## Methods

### Ethics statement

No specific permits were required to collect and study the clam from the described fields. They are not under an endangered or protected list and thus have no control over the collection of samples. The experimental protocols used to conduct this study were approved by the Institutional Animal Ethical Committee of the ICAR Central Marine Fisheries Research Institute (CMFRI), Kochi. Additionally, the methodologies utilized were following the guidelines outlined in ARRIVE (Animal Research: Reporting of *In Vivo* Experiments) guidelines available at http://arriveguidelines.org.

### Study area and sample collection

**Fig. 1:**
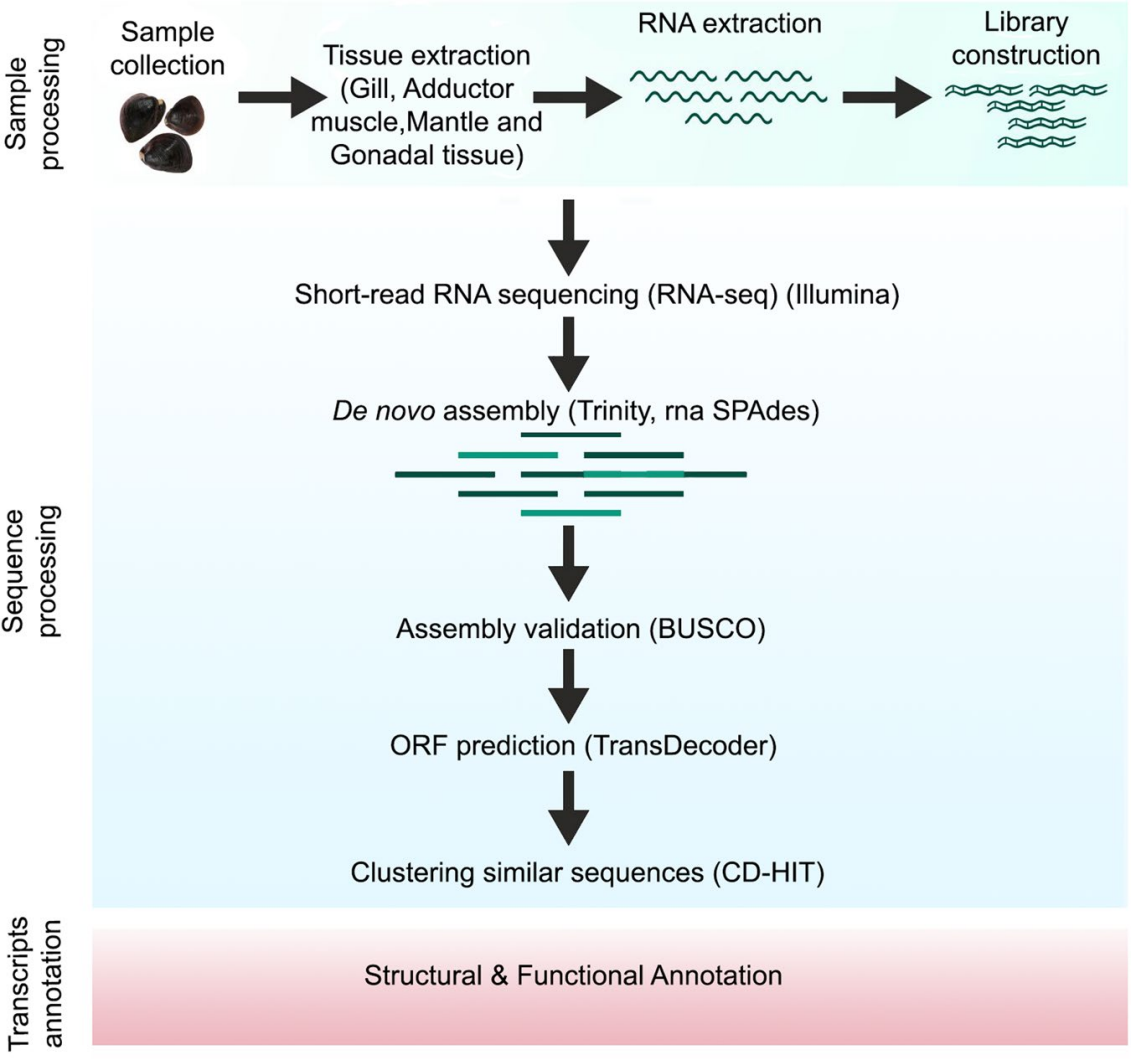
Workflow of the bioinformatic pipeline, from raw data to annotated transcripts for the *de novo* transcriptome assembly of *Villorita cyprinoides*.

The healthy adult black clam specimens with a shell length (SL) of 30 ± 3.5 mm and a total body weight (BW) of 35–40 grams were collected using *kolli* (an indigenous hand rake net) from Vembanad Lake, a Ramsar site in Kerala, India in March 2021. The study locations in Vembanad Lake included the southern dominant freshwater region, Muhamma: 9°36′18.6″N 76°22′01.2″E and the northern saltwater region, Vaikom: 9°44′35.8″N 76°23′03.4″E, divided seasonally by the Thaneermukkom barrage^10^. Individuals were primarily identified using malacological literature^26^. A single specimen from each sampling site was randomly selected and tissue samples of the gill, foot, adductor muscle, mantle, and gonad were dissected and stored separately in RNA later (Sigma). The remaining tissue samples were fixed in absolute ethanol for species confirmation by the molecular method. The tubes were transported to the laboratory at 4 °C and kept at −80 °C until RNA extraction. An overview of the workflow is shown in Fig. 1.

### DNA extraction and barcoding

Extraction of total genomic DNA from ethanol-preserved tissue was carried out using the Qiagen DNeasy Blood and Tissue Kit (QIAGEN, Valencia, CA, USA) and the barcode region of mitochondrial cytochrome c oxidase subunit I (COI) was amplified following the PCR protocol: initial denaturation for 5 min at 94 °C followed by 35 cycles of 30 s at 94 °C, 30 s at 42 °C, and 1 min at 72 °C with a final extension of 5 minutes at 72 °C, using the primer set LCO1490 /HCO2198^27^. The amplified PCR products were purified, bi-directionally sequenced, edited, and aligned in MEGA 11^28^. The COI sequences generated were nBLAST against the GenBank database to confirm species identification.

### RNA extraction and mRNA library preparation and sequencing

Total RNA from each tissue sample (gill, foot, adductor muscle, mantle, and gonad tissues from each clam) was extracted, purified, and quantified separately. RNA isolation was carried out using TRIzol Reagent (Invitrogen) with the manufacturer’s instructions and treated with RNase-free DNase I (TaKaRa). The quality and quantity of isolated RNA were confirmed using 0.8% denaturing agarose gel, Qubit Fluorometer 3.0 (ThermoFisher), and Agilent 4200 Bioanalyzer (Agilent Technologies, USA). 1.5 µg of RNA from the five tissues of each clam were pooled in equimolar concentration to prepare two RNA-seq libraries (VcypMt2, VcypVt2) using the TruSeq Standard mRNA-seq library preparation kit following the manufacturer’s protocol (Illumina, USA). Barcoded libraries were then sequenced on the Illumina NovaSeq 6000 platform with 150 base pair (bp) PE mode.

### *De novo* transcriptome assembly, refinement, and quality assessment

Illumina sequences generated from two black clam specimens were processed separately and default settings were used for all software analyses unless otherwise stated. Primarily, FastQC (http://www.bioinformatics.babraham.ac.uk/projects/fastqc/) was used to assess quality per base, overexpressed sequences, and adapter content from sequenced raw reads. The adapters and poly-A tails were trimmed and ambiguous reads were removed, N > 5% from both trailing and leading sequences. High-quality reads with a Phred score >30, and a minimum read length >50 bp were retained using Trimmomatic v0.35^29^. D*e novo* transcriptome assemblies of clean reads were performed using Trinity v2.20 and rnaSPAdes v3.9 with a *k-mer* of 25^30,31^. Redundancies from the primary assembled transcriptomes were removed by CD-HIT v4.6.5^32^.

Bowtie v2.3.5 and the Benchmark Universal Single Copy Orthologs (BUSCO) software were used to reassess the quality and completeness of the assembled transcriptomes^33,34^. In order to determine the degree of representation of reads within the transcriptome assemblies, Bowtie mapped back the Illumina RNA-seq reads to the corresponding transcriptome assemblies. The alignment parameters included end-to-end sensitivity, and a maximum number of mismatches, N = 1. The percentage of single-copy orthologous genes in the *de novo* assemblies was determined by BUSCO, which was compared to the mollusca_odb10 datasets.

### Functional annotation

Open Reading Frames (ORFs) and coding regions from non-redundant transcripts were predicted by TransDecoder v3.0.0 with default parameters^35^. The predicted protein coding regions were then searched for homology using BLASTtx and BLASTp against NCBInr (https://www.ncbi.nlm.nih.gov/), UniProtKB (https://www.ebi.ac.uk/uniprot/), RefSeq (https://www.ncbi.nlm.nih.gov/refseq/) and Pfam databases with an e-value threshold of 1e-5^36,37^. Gene Ontology (GO) annotations were also performed using EggNOG, KEGG, and Metascape (https://metascape.org/), which assigns transcripts to cellular components, cellular functions, and biological processes^38^. Subsequently, functional protein domains were then assigned using InterProScan v4.0^39^.

### Repetitive sequence identification

GMATA (Genome-wide Microsatellite Analyzing Towards Application) was used to identify simple sequence repeats (SSR) with a length of 2–10 bp and a repeat of 5 in the generated transcriptome^40^.

### Data analysis and visualization

The data analysis and visualization were performed via R software using the packages *ggplot2, tidyr, dplyr, stringi, plotrix, forcats, ggVennDiagram, venn, ggpolypath*, and *RColorBrewer*^41^.

## Data Records

The COI amplicons amplified from morphologically identified specimens were sequenced by Sanger sequencing and were submitted to GenBank with accession numbers OP999653^42^ and OP999654^43^. All the Illumina sequencing reads were submitted to NCBI-SRA (National Centre for Biotechnology Information-Sequence Read Archive) under Bioproject ID: PRJNA910160 with accession numbers SRR22577462^44^ and SRR22577463^45^. The corresponding BioSample IDs are SAMN32114842 and SAMN32114843. The transcriptome assembly and annotations of *V. cyprinoides* were shared through the Figshare platform^46^.

## Technical Validation

**Fig. 2:**
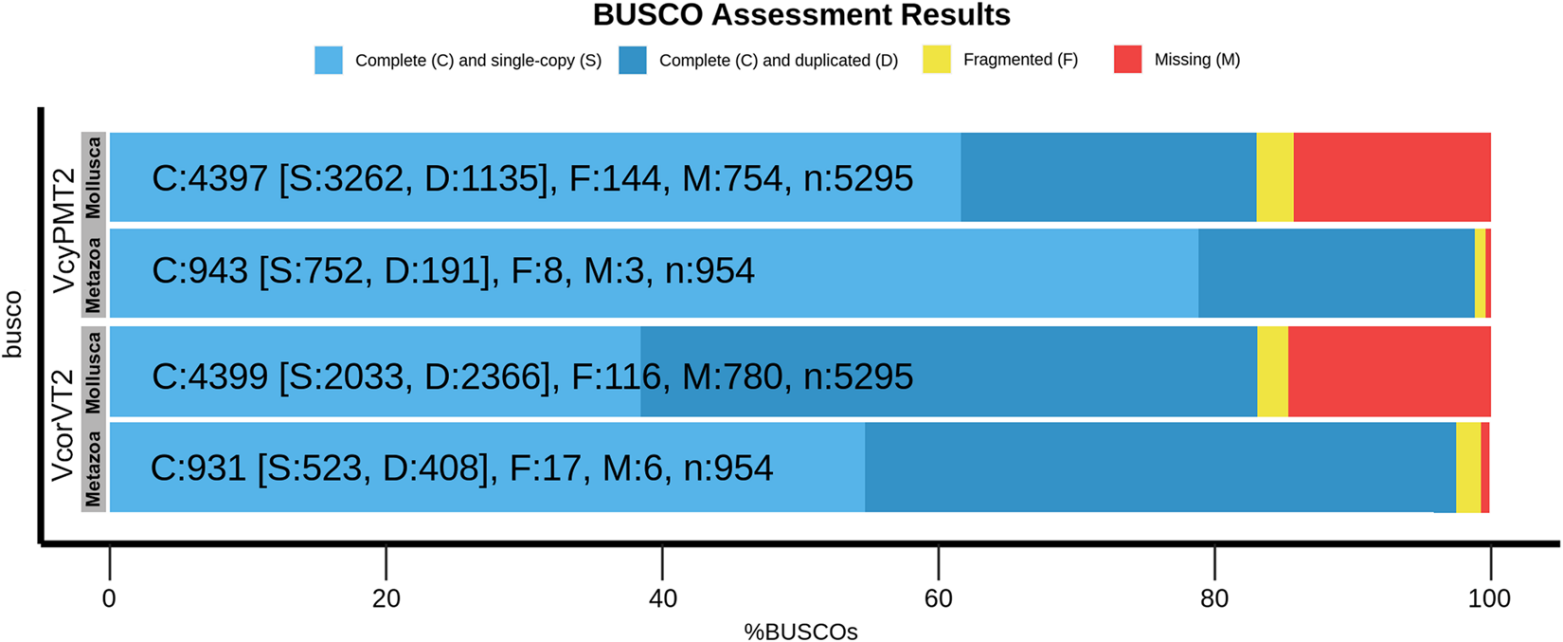
Summarized benchmarking of BUSCO notations for assessing the completeness of transcriptome assemblies with Mollusca_obd10, S: complete and single-copy BUSCOs; D: complete and duplicated BUSCOs; F: fragmented BUSCOs; M: missing BUSCOs. The different colors represent different kinds of BUSCOs, accordingly. The numbers indicate the percentage of different kinds of BUSCOs within the total number of BUSCO groups searched.

**Fig. 3:**
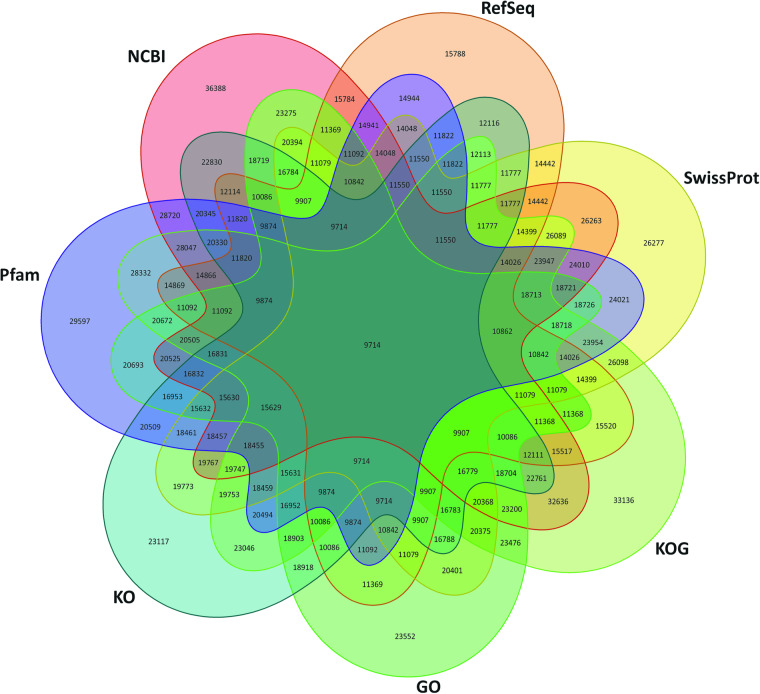
Venn diagram of genes and predicted proteins aligned to different databases. NCBI– non-redundant protein database, RefSeq–NCBI Reference Sequence database, SwissProt, KOG–EuKaryotic Orthologous Groups database, GO–Gene Ontology, KO–KEGG Orthology, and Pfam–The protein families database. A total of 9714 transcripts were annotated across all six databases.

**Fig. 4:**
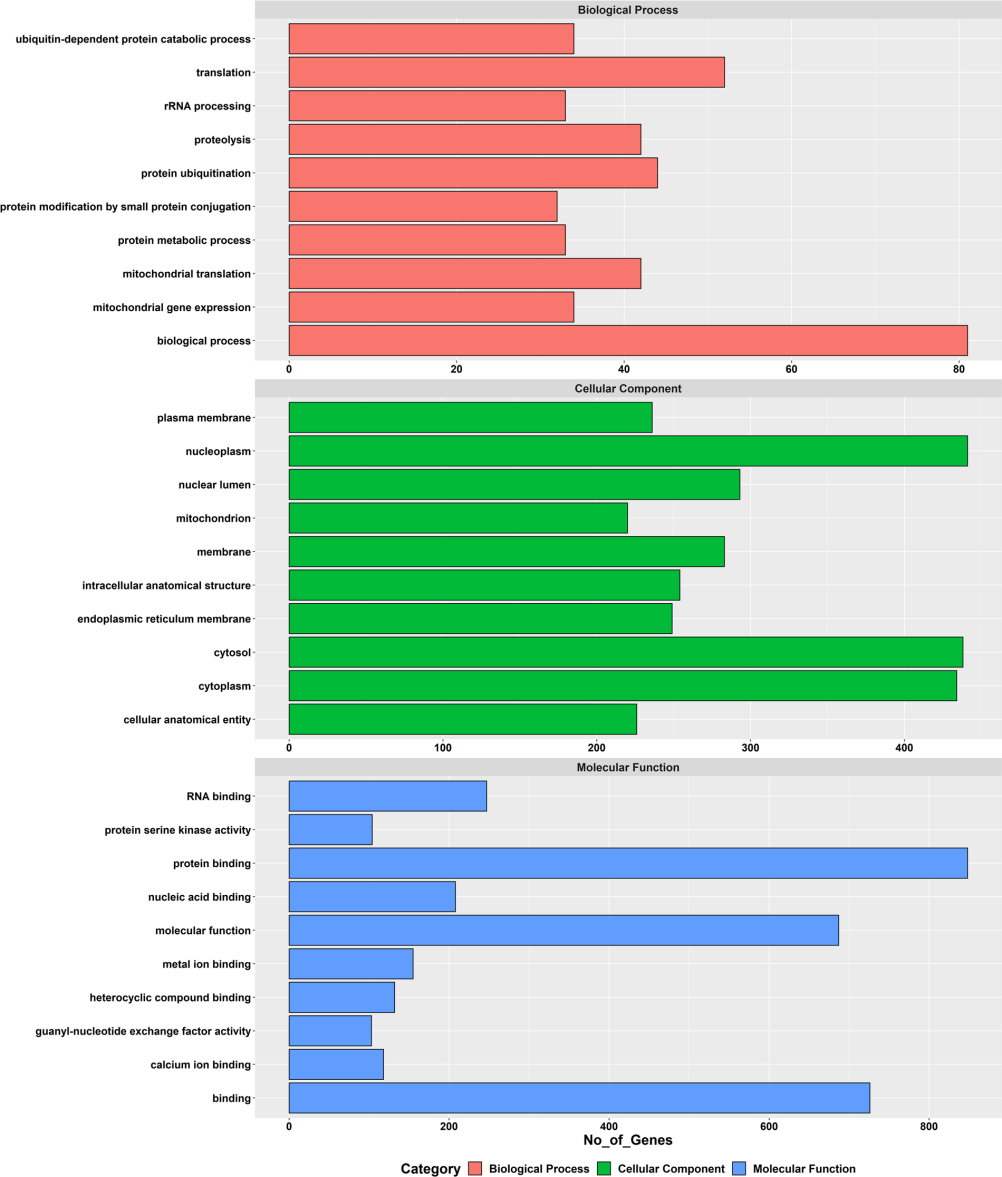
Bar Chart of Gene Ontology (GO) classification. The findings were categorized and summarized into three primary categories: cellular component, molecular function, and biological process.

**Fig. 5:**
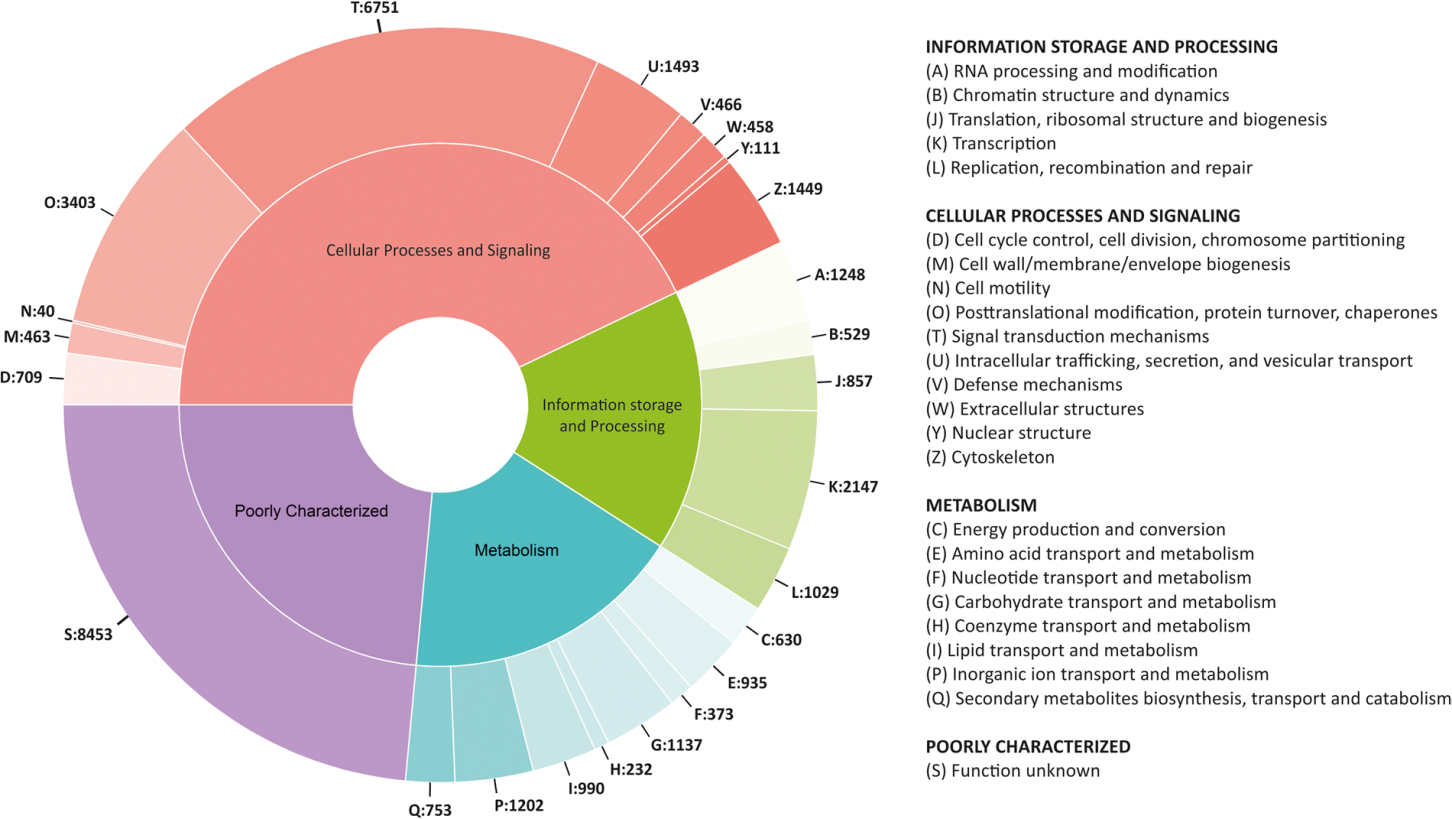
Gene functional classifications in the EuKaryotic Orthologous Groups (KOG) database showing the categorization of genes into 24 functional classes (A–Q, R-W, X- Z). The numbers assigned next to the function categories refer to the relative share of the categories.

**Fig. 6:**
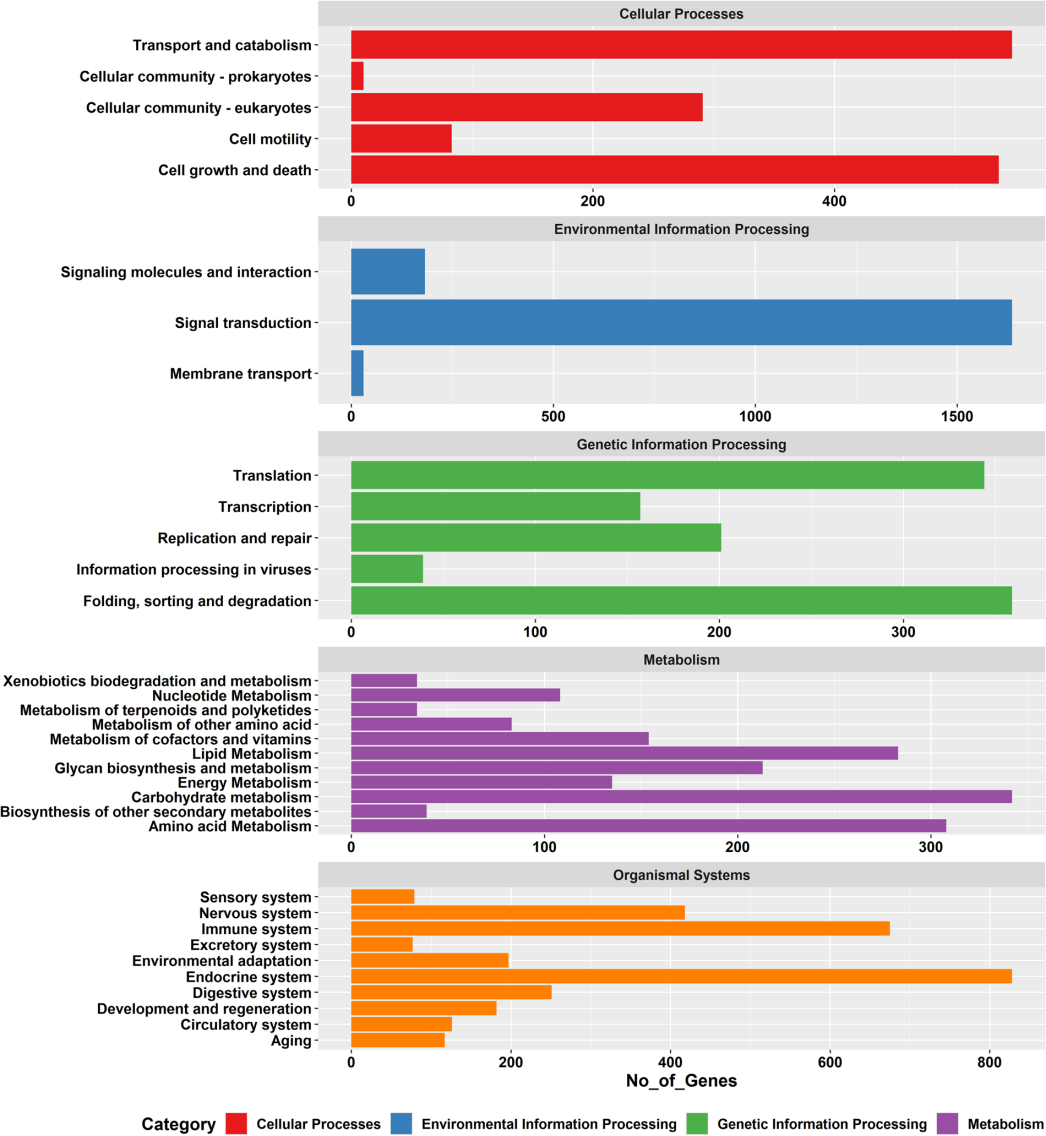
KEGG pathway classification map. Genes were divided into five branches according to the biological pathways in which they participated: Cellular Processes, Environmental Information Processing, Genetic Information Processing, Metabolism, and Organismal Systems. The number of annotated genes is represented on the X-axis, and KEGG categories are shown on the Y-axis.

**Fig. 7:**
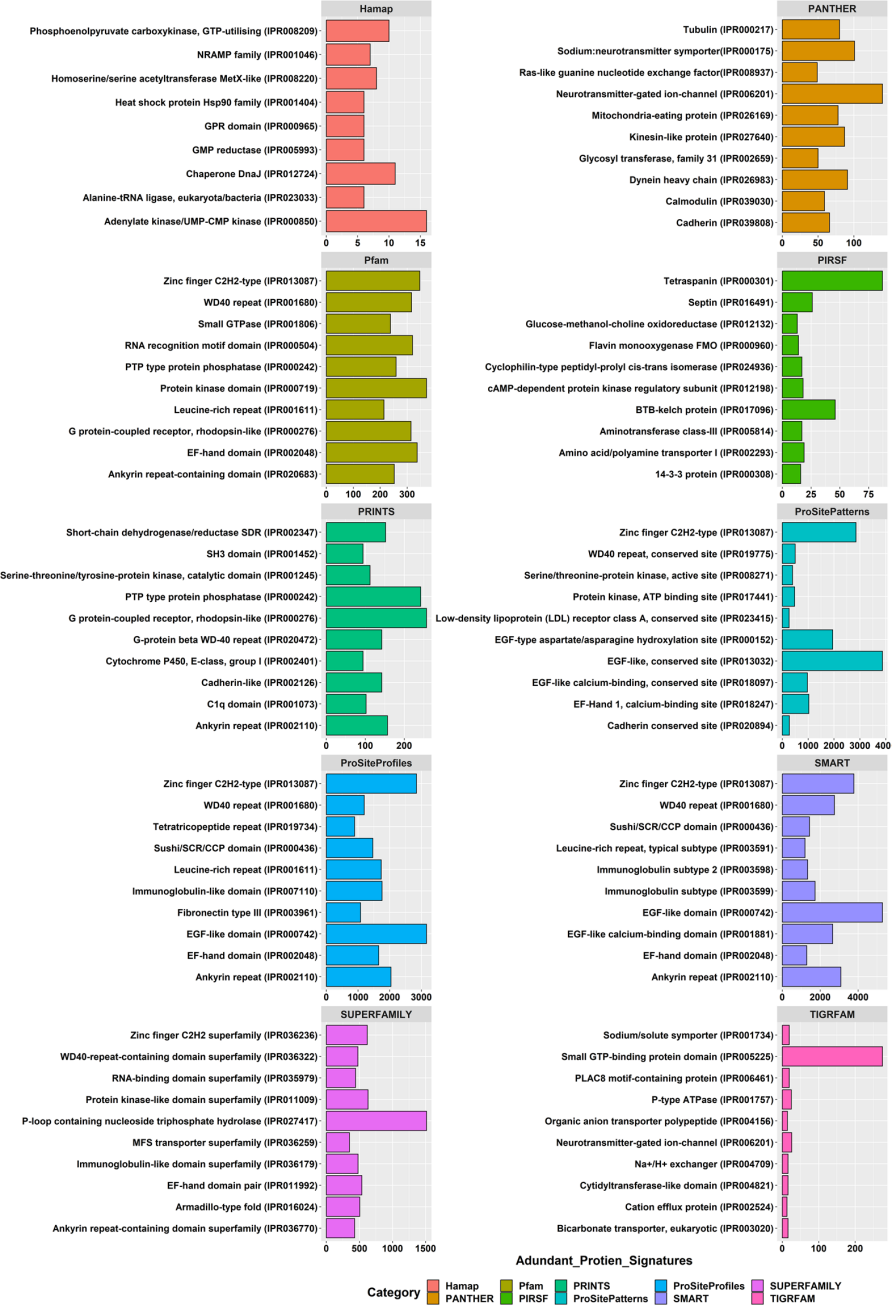
An overview of InterProScan annotation. The top 10 most abundant protein signatures assigned by different member databases through InterProScan (IPS) are shown. The X-axis represents the number of genes associated with the respective protein signatures and the Y-axis represents their abundance.

**Fig. 8:**
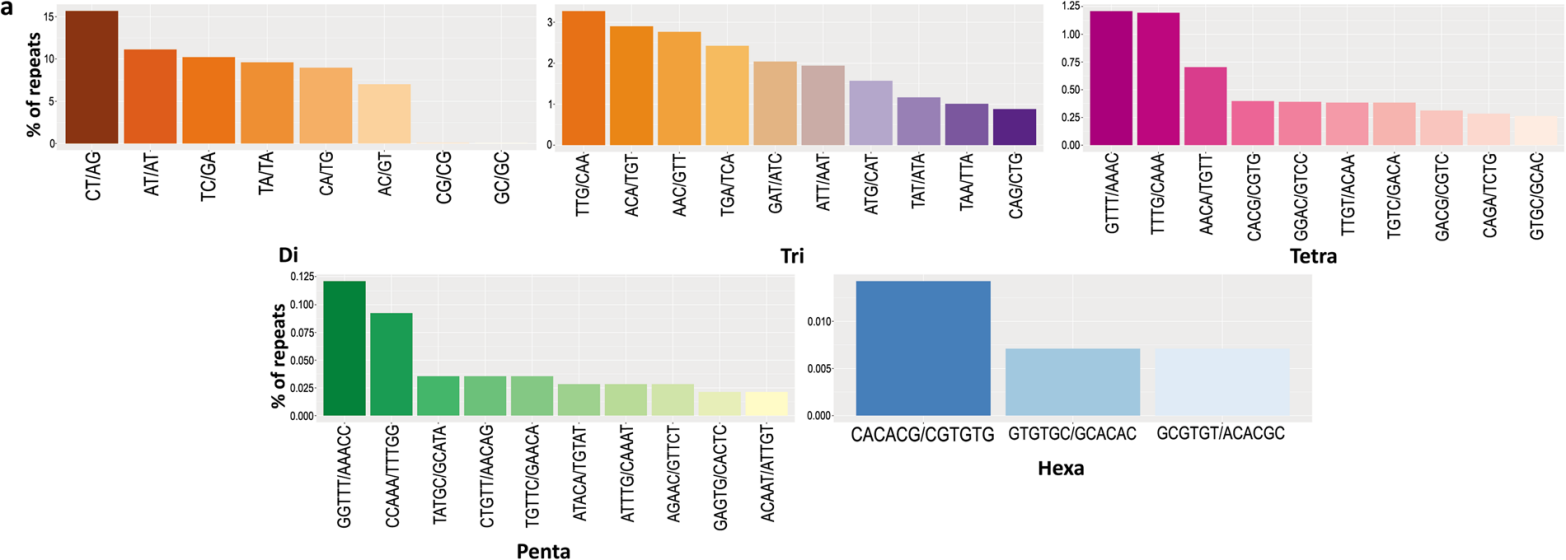
The figure illustrates the percentage distribution of di-, tri-, tetra-, penta-, and hexanucleotide repeat motifs identified in the transcriptome assembly of *Villorita cyprinoides* using GMATA software. The most common simple sequence repeat (SSR) motif identified was the CT/AG dinucleotide repeat.

The RNA isolated from different tissues with prominent and consistent 18S rRNA bands was used for library preparation. After two Illumina paired-end sequencing and quality filtering, the refrained high-quality clean transcripts were used to reconstruct transcriptomes using the reference-free *de novo* transcriptome assembly method. The quality filtering approach to remove redundancies in transcriptome assemblies was done by the elimination of repetitive regions, contigs, and singletons by CD-HIT. Comparison of statistics of *de novo* transcriptome assemblies using Trinity and rnaSPAdes assemblers is shown in Table 1. Conversely, the completeness of final assemblies was evaluated using BUSCO, and the percentage of single copies, missing, and fragmented BUSCO groups are depicted in Fig. 2. The transcripts annotated from Coding Domain Sequences (CDSs) identified through the six databases– UniProtKB, NCBI, Pfam, KOG, KEGG, and RefSeq are shown in Fig. 3. In addition, statistics of functional annotation towards different databases are summarized in Table 2. The categorization of GO terminologies such as biological process (BP), molecular function (MF), and cellular components (CC) is shown in Fig. 4. Besides, the KOG (EuKaryotic Orthologous Groups) functional classification groups the transcripts into 24 KOG categories, as depicted in Fig. 5, while Fig. 6 shows the transcripts mapped to five KEGG pathways. In addition, the ten most common protein domains obtained from the transcripts using InterPro member databases; Hamap, Pfam, Prints, ProSiteProfiles, SUPERFAMILY, PANTHER, PIRSF, ProSitePatterns, SMART, TIGRFAM are depicted in Fig. 7. Among the detected SSRs, the frequencies of di-, tri-, tetra-, penta-, and hexanucleotides were 62.7%, 26.7%, 9.5%, 0.89% and 0.02% respectively (Fig. 8, Tables S1–S3). From the data, the most prevalent di- and trinucleotide repeat was also detected (Table S4). These datasets expand indispensable transcriptomic resources for future research on functional genomics, gene characterization, and expression profiling in *V. cyprinoides*, as well as expanding molecular data for evolutionary studies in the family Cyrenidae.

**Table 1:** Statistics of *de novo* assemblies and quality filtering of *Villorita cyprinoides* transcriptomes were displayed.

Assembly Statistics	TRINITY		rnaSPAdes	
	VcypVt2	VcypMt2	VcypVt2	VcypMt2
Assembly size (bp)	177139464	183586011	127649071	133110033
Total number of contigs (bp)	142491	154847	112588	120855
N50 (bp)	2588	2461	2618	2517
Mean contig length (bp)	1243	1185	1133	1101
Longest contig (bp)	27080	31762	29464	35015
GC content (%)	38.14	37.96	37.88	37.76
**Filtering Statistics**	VcypVt2	VcypMt2	VcypVt2	VcypMt2
Assembly size (bp)	176775746	124858206	117467820	122551596
Total number of contigs (bp)	142370	127309	104403	112260
N50 (bp)	2585	2588	2574	2470
Mean contig length (bp)	1241	1243	1125	1090
Longest contig (bp)	27080	31762	29464	35015
GC content (%)	38.14	38.14	37.90	37.76

**Table 2:** Statistics of functional annotation by different databases.

Databases	Number of genes annotated	Percentage
NCBI	18795	39%
UniPortKB	23026	48%
RefSeq	15788	33%
Pfam	29597	62%
GO	23552	49%
KOG	33136	69%
KEGG	9098	19%

### Supplementary information

Details of single sequence repeats (SSRs) identified using GMATA

## Data Availability

No custom code was generated in this study. The bioinformatics tools implemented in this study and their versions, settings, and parameters were explained in the Methods section. In cases where no specific settings were specified for a particular tool, default parameters were employed.
