# Supplementary material for: Tidings from the Tides–De novo transcriptome assembly of the endemic estuarine bivalve Villorita cyprinoides
Source: Sci Data. 2024 Jul 2;11:723. doi: 10.1038/s41597-024-03541-4 (PMC11219770; doi:10.1038/s41597-024-03541-4)
Supplement: Supplementary file 1 — Details of single sequence repeats (SSRs) identified using GMATA [file 41597_2024_3541_MOESM1_ESM.docx]

**Tidings from the Tides–*De novo* transcriptome assembly of the endemic estuarine bivalve *Villorita cyprinoides***

Summaya Rahuman^1,3^, Jeena N. S^1*^, Wilson Sebastian^2^, Eldho Varghese^1^, and Asokan P. K^1^.

^1^Indian Council of Agricultural Research - Central Marine Fisheries Research Institute, Kochi, 682 018, Kerala, India.

^2^Centre for Marine Living Resources and Ecology, Kochi, 682508, Kerala, India.

^3^Mangalore University, Mangalagangotri, Mangalore, 574 199, Karnataka, India.

^*^Corresponding author

*Email address*: [jeena.ns@icar.gov.in](mailto:jeena.ns@icar.gov.in)

| Supplementary Table 1: SSR repeat count and repeat number recognized by GMATA | | |
| --- | --- | --- |
| **Motif(-mer)** | **Total** | **Percentage** |
| 2 | 8828 | 62.7746569 |
| 3 | 3766 | 26.77949228 |
| 4 | 1337 | 9.507217521 |
| 5 | 126 | 0.895968143 |
| 6 | 4 | 0.028443433 |
| 9 | 1 | 0.007110858 |
| 10 | 1 | 0.007110858 |
| Total above | Total above | Total above |
| 7 | 14063 | 100 |

| Supplementary Table 2: SSR motifs and total number recognized by GMATA | | |
| --- | --- | --- |
| **Motif** | **Total** | **Percentage** |
| AT | 1564 | 11.12138235 |
| TA | 1349 | 9.592547821 |
| CT | 1187 | 8.440588779 |
| AG | 1018 | 7.23885373 |
| TC | 867 | 6.165114129 |
| TG | 631 | 4.486951575 |
| CA | 629 | 4.472729858 |
| AC | 596 | 4.238071535 |
| GA | 569 | 4.046078362 |
| GT | 388 | 2.759013013 |
| AAC | 283 | 2.012372893 |
| TTG | 281 | 1.998151177 |
| TGT | 234 | 1.663940838 |
| TCA | 186 | 1.32261964 |
| CAA | 179 | 1.272843632 |
| ACA | 174 | 1.237289341 |
| TGA | 155 | 1.102183033 |
| GAT | 144 | 1.023963592 |
| ATC | 143 | 1.016852734 |
| AAT | 138 | 0.981298443 |
| ATT | 135 | 0.959965868 |
| ATG | 116 | 0.824859561 |
| ATA | 111 | 0.789305269 |
| GTT | 106 | 0.753750978 |
| CAT | 105 | 0.746640119 |
| AAAC | 103 | 0.732418403 |
| TTA | 95 | 0.675531537 |
| TTTG | 89 | 0.632866387 |
| CAAA | 79 | 0.561757804 |
| ACT | 77 | 0.547536088 |
| TGTT | 70 | 0.49776008 |
| GTTT | 67 | 0.476427505 |
| CAG | 63 | 0.447984072 |
| TGC | 63 | 0.447984072 |
| CTG | 61 | 0.433762355 |
| AAG | 58 | 0.41242978 |
| CTA | 55 | 0.391097205 |
| TAT | 53 | 0.376875489 |
| TCT | 51 | 0.362653772 |
| TAA | 47 | 0.334210339 |
| AGT | 46 | 0.327099481 |
| CCA | 44 | 0.312877764 |
| TTC | 40 | 0.284434331 |
| GACG | 40 | 0.284434331 |
| TGG | 37 | 0.263101756 |
| CTT | 36 | 0.255990898 |
| GCA | 35 | 0.24888004 |
| CACG | 34 | 0.241769182 |
| TAC | 34 | 0.241769182 |
| TGTC | 34 | 0.241769182 |
| GGAC | 34 | 0.241769182 |
| TCC | 33 | 0.234658323 |
| TTGT | 33 | 0.234658323 |
| GAA | 32 | 0.227547465 |
| CAC | 32 | 0.227547465 |
| AACA | 29 | 0.20621489 |
| ACC | 27 | 0.191993174 |
| AGA | 26 | 0.184882315 |
| AGC | 25 | 0.177771457 |
| GTGC | 24 | 0.170660599 |
| GTG | 22 | 0.156438882 |
| CGTG | 22 | 0.156438882 |
| GTCC | 21 | 0.149328024 |
| TAG | 21 | 0.149328024 |
| ACAA | 21 | 0.149328024 |
| GGT | 21 | 0.149328024 |
| GCT | 20 | 0.142217166 |
| GACA | 20 | 0.142217166 |
| TCTG | 20 | 0.142217166 |
| CAGA | 20 | 0.142217166 |
| TTTA | 18 | 0.127995449 |
| AAAT | 17 | 0.120884591 |
| GGA | 16 | 0.113773732 |
| CGCA | 16 | 0.113773732 |
| CG | 16 | 0.113773732 |
| GAG | 16 | 0.113773732 |
| GTA | 16 | 0.113773732 |
| CTC | 15 | 0.106662874 |
| CCT | 14 | 0.099552016 |
| GC | 14 | 0.099552016 |
| GCAC | 13 | 0.092441158 |
| ATCA | 13 | 0.092441158 |
| CGGA | 13 | 0.092441158 |
| ACG | 13 | 0.092441158 |
| AACC | 12 | 0.085330299 |
| TGTA | 12 | 0.085330299 |
| CTGT | 12 | 0.085330299 |
| TGCG | 11 | 0.078219441 |
| AATA | 11 | 0.078219441 |
| TAAA | 11 | 0.078219441 |
| GCGT | 10 | 0.071108583 |
| AGAC | 10 | 0.071108583 |
| ATAA | 10 | 0.071108583 |
| TGGT | 10 | 0.071108583 |
| AAACC | 10 | 0.071108583 |
| GTCT | 10 | 0.071108583 |
| ATTG | 9 | 0.063997725 |
| AACG | 9 | 0.063997725 |
| TCAA | 9 | 0.063997725 |
| TTAT | 9 | 0.063997725 |
| ACGC | 9 | 0.063997725 |
| TTTGG | 9 | 0.063997725 |
| ATTT | 9 | 0.063997725 |
| TCCG | 9 | 0.063997725 |
| TGAT | 9 | 0.063997725 |
| GTTC | 9 | 0.063997725 |
| TATT | 8 | 0.056886866 |
| TGAG | 8 | 0.056886866 |
| AAGC | 8 | 0.056886866 |
| TCG | 8 | 0.056886866 |
| ACAG | 8 | 0.056886866 |
| GACC | 8 | 0.056886866 |
| AATT | 7 | 0.049776008 |
| GGTC | 7 | 0.049776008 |
| GAC | 7 | 0.049776008 |
| GGTTT | 7 | 0.049776008 |
| AAAG | 6 | 0.04266515 |
| CGT | 6 | 0.04266515 |
| TCAG | 6 | 0.04266515 |
| TGAA | 6 | 0.04266515 |
| TGTTC | 5 | 0.035554291 |
| TTAA | 5 | 0.035554291 |
| TTGG | 5 | 0.035554291 |
| TGAC | 5 | 0.035554291 |
| GAAC | 5 | 0.035554291 |
| AATC | 5 | 0.035554291 |
| CGAC | 5 | 0.035554291 |
| CTGTT | 5 | 0.035554291 |
| CTCA | 5 | 0.035554291 |
| CGTT | 5 | 0.035554291 |
| GTTG | 5 | 0.035554291 |
| CAAC | 5 | 0.035554291 |
| TATGC | 5 | 0.035554291 |
| CATC | 4 | 0.028443433 |
| GATC | 4 | 0.028443433 |
| GATG | 4 | 0.028443433 |
| CGTC | 4 | 0.028443433 |
| ATTA | 4 | 0.028443433 |
| ATTTG | 4 | 0.028443433 |
| CCAAA | 4 | 0.028443433 |
| CCTC | 4 | 0.028443433 |
| GGTT | 4 | 0.028443433 |
| GTC | 4 | 0.028443433 |
| ATACA | 4 | 0.028443433 |
| CATA | 4 | 0.028443433 |
| GTCA | 4 | 0.028443433 |
| CCAA | 4 | 0.028443433 |
| TCGG | 3 | 0.021332575 |
| CTCC | 3 | 0.021332575 |
| CAAT | 3 | 0.021332575 |
| CGA | 3 | 0.021332575 |
| TTTC | 3 | 0.021332575 |
| CAGG | 3 | 0.021332575 |
| TGGA | 3 | 0.021332575 |
| TCCA | 3 | 0.021332575 |
| GTATT | 3 | 0.021332575 |
| AGG | 3 | 0.021332575 |
| GAAG | 3 | 0.021332575 |
| TAAT | 3 | 0.021332575 |
| GAGT | 3 | 0.021332575 |
| GAGTG | 3 | 0.021332575 |
| TCGT | 3 | 0.021332575 |
| CGGT | 3 | 0.021332575 |
| TACA | 3 | 0.021332575 |
| TATG | 3 | 0.021332575 |
| GATT | 3 | 0.021332575 |
| AGTG | 3 | 0.021332575 |
| AAACA | 2 | 0.014221717 |
| GTTCT | 2 | 0.014221717 |
| GAAA | 2 | 0.014221717 |
| GTGTG | 2 | 0.014221717 |
| GACTA | 2 | 0.014221717 |
| GAGC | 2 | 0.014221717 |
| ACCT | 2 | 0.014221717 |
| TACC | 2 | 0.014221717 |
| ACGG | 2 | 0.014221717 |
| ACAT | 2 | 0.014221717 |
| CCGA | 2 | 0.014221717 |
| TAACA | 2 | 0.014221717 |
| GTCG | 2 | 0.014221717 |
| TTCT | 2 | 0.014221717 |
| TTCTG | 2 | 0.014221717 |
| GTTTG | 2 | 0.014221717 |
| TCCT | 2 | 0.014221717 |
| ATGG | 2 | 0.014221717 |
| TTAC | 2 | 0.014221717 |
| CATT | 2 | 0.014221717 |
| ACCG | 2 | 0.014221717 |
| TGGTT | 2 | 0.014221717 |
| TTAG | 2 | 0.014221717 |
| TTGA | 2 | 0.014221717 |
| TGCAG | 2 | 0.014221717 |
| AGAAC | 2 | 0.014221717 |
| TTTGT | 2 | 0.014221717 |
| TTGTG | 2 | 0.014221717 |
| TCAC | 2 | 0.014221717 |
| CCTCC | 2 | 0.014221717 |
| CACACG | 2 | 0.014221717 |
| AGAA | 2 | 0.014221717 |
| TCCC | 2 | 0.014221717 |
| GGAG | 2 | 0.014221717 |
| ATAC | 2 | 0.014221717 |
| TTTTG | 2 | 0.014221717 |
| ACTTA | 2 | 0.014221717 |
| CTGC | 2 | 0.014221717 |
| TTCG | 2 | 0.014221717 |
| TTGTA | 2 | 0.014221717 |
| ATTGT | 2 | 0.014221717 |
| ACAGT | 1 | 0.007110858 |
| GTACG | 1 | 0.007110858 |
| TCGC | 1 | 0.007110858 |
| ACCA | 1 | 0.007110858 |
| AATTC | 1 | 0.007110858 |
| GCAG | 1 | 0.007110858 |
| TTCGT | 1 | 0.007110858 |
| TAGA | 1 | 0.007110858 |
| TGCCC | 1 | 0.007110858 |
| ATTC | 1 | 0.007110858 |
| TGTAG | 1 | 0.007110858 |
| GGCA | 1 | 0.007110858 |
| TAGT | 1 | 0.007110858 |
| CGAA | 1 | 0.007110858 |
| CACT | 1 | 0.007110858 |
| ACTG | 1 | 0.007110858 |
| CCG | 1 | 0.007110858 |
| AGGT | 1 | 0.007110858 |
| CAGAT | 1 | 0.007110858 |
| TTCTA | 1 | 0.007110858 |
| TTATC | 1 | 0.007110858 |
| GTTTA | 1 | 0.007110858 |
| CTTT | 1 | 0.007110858 |
| AGTGGCCTT | 1 | 0.007110858 |
| AAGA | 1 | 0.007110858 |
| CAATC | 1 | 0.007110858 |
| GGCGT | 1 | 0.007110858 |
| TACG | 1 | 0.007110858 |
| CTAC | 1 | 0.007110858 |
| ACATC | 1 | 0.007110858 |
| GTAAT | 1 | 0.007110858 |
| ACCAA | 1 | 0.007110858 |
| AACAT | 1 | 0.007110858 |
| AGTA | 1 | 0.007110858 |
| CCAG | 1 | 0.007110858 |
| GTGAA | 1 | 0.007110858 |
| CCGT | 1 | 0.007110858 |
| ACAAT | 1 | 0.007110858 |
| ATGGT | 1 | 0.007110858 |
| CTTG | 1 | 0.007110858 |
| AACAC | 1 | 0.007110858 |
| ATATTCATGC | 1 | 0.007110858 |
| TCTGT | 1 | 0.007110858 |
| ATCC | 1 | 0.007110858 |
| GCGTGT | 1 | 0.007110858 |
| TGCT | 1 | 0.007110858 |
| AATG | 1 | 0.007110858 |
| ACTA | 1 | 0.007110858 |
| TGTGC | 1 | 0.007110858 |
| CGAG | 1 | 0.007110858 |
| AACT | 1 | 0.007110858 |
| ATCG | 1 | 0.007110858 |
| CCAT | 1 | 0.007110858 |
| TATCG | 1 | 0.007110858 |
| GTAAA | 1 | 0.007110858 |
| TGGC | 1 | 0.007110858 |
| AGCAC | 1 | 0.007110858 |
| TTGGT | 1 | 0.007110858 |
| CATAA | 1 | 0.007110858 |
| GGTGT | 1 | 0.007110858 |
| CACGA | 1 | 0.007110858 |
| ATGAC | 1 | 0.007110858 |
| AACAA | 1 | 0.007110858 |
| GGGA | 1 | 0.007110858 |
| TCTTG | 1 | 0.007110858 |
| GTGTGC | 1 | 0.007110858 |
| GCCAC | 1 | 0.007110858 |
| CTGG | 1 | 0.007110858 |
| TATC | 1 | 0.007110858 |
| Total above | Total above | Total above |
| 277 | 14063 | 100 |

| Supplementary Table 3: SSR grouped motifs and total number recognized by GMATA | | |
| --- | --- | --- |
| **Grouped Motif** | **Total** | **Percentage** |
| CT/AG | 2205 | 15.67944251 |
| AT/AT | 1564 | 11.12138235 |
| TC/GA | 1436 | 10.21119249 |
| TA/TA | 1349 | 9.592547821 |
| CA/TG | 1260 | 8.959681434 |
| AC/GT | 984 | 6.997084548 |
| TTG/CAA | 460 | 3.270994809 |
| ACA/TGT | 408 | 2.901230178 |
| AAC/GTT | 389 | 2.766123871 |
| TGA/TCA | 341 | 2.424802674 |
| GAT/ATC | 287 | 2.040816327 |
| ATT/AAT | 273 | 1.941264311 |
| ATG/CAT | 221 | 1.57149968 |
| GTTT/AAAC | 170 | 1.208845908 |
| TTTG/CAAA | 168 | 1.194624191 |
| TAT/ATA | 164 | 1.166180758 |
| TAA/TTA | 142 | 1.009741876 |
| CAG/CTG | 124 | 0.881746427 |
| ACT/AGT | 123 | 0.874635569 |
| AACA/TGTT | 99 | 0.70397497 |
| GCA/TGC | 98 | 0.696864111 |
| AAG/CTT | 94 | 0.668420678 |
| CCA/TGG | 81 | 0.575979521 |
| TCT/AGA | 77 | 0.547536088 |
| CTA/TAG | 76 | 0.540425229 |
| TTC/GAA | 72 | 0.511981796 |
| CACG/CGTG | 56 | 0.398208064 |
| GGAC/GTCC | 55 | 0.391097205 |
| TTGT/ACAA | 54 | 0.383986347 |
| GTG/CAC | 54 | 0.383986347 |
| TGTC/GACA | 54 | 0.383986347 |
| TAC/GTA | 50 | 0.355542914 |
| GGA/TCC | 49 | 0.348432056 |
| GGT/ACC | 48 | 0.341321197 |
| AGC/GCT | 45 | 0.319988623 |
| GACG/CGTC | 44 | 0.312877764 |
| CAGA/TCTG | 40 | 0.284434331 |
| GTGC/GCAC | 37 | 0.263101756 |
| CTC/GAG | 31 | 0.220436607 |
| TAAA/TTTA | 29 | 0.20621489 |
| TGCG/CGCA | 27 | 0.191993174 |
| ATTT/AAAT | 26 | 0.184882315 |
| CGGA/TCCG | 22 | 0.156438882 |
| TGAT/ATCA | 22 | 0.156438882 |
| ACAG/CTGT | 20 | 0.142217166 |
| GTCT/AGAC | 20 | 0.142217166 |
| TTAT/ATAA | 19 | 0.135106307 |
| AATA/TATT | 19 | 0.135106307 |
| GCGT/ACGC | 19 | 0.135106307 |
| CGT/ACG | 19 | 0.135106307 |
| GGTTT/AAACC | 17 | 0.120884591 |
| CCT/AGG | 17 | 0.120884591 |
| CG/CG | 16 | 0.113773732 |
| GGTT/AACC | 16 | 0.113773732 |
| GACC/GGTC | 15 | 0.106662874 |
| TACA/TGTA | 15 | 0.106662874 |
| CGTT/AACG | 14 | 0.099552016 |
| GC/GC | 14 | 0.099552016 |
| GAAC/GTTC | 14 | 0.099552016 |
| CCAAA/TTTGG | 13 | 0.092441158 |
| CTCA/TGAG | 13 | 0.092441158 |
| ATTG/CAAT | 12 | 0.085330299 |
| GTC/GAC | 11 | 0.078219441 |
| TCG/CGA | 11 | 0.078219441 |
| TTGA/TCAA | 11 | 0.078219441 |
| ACCA/TGGT | 11 | 0.078219441 |
| CAAC/GTTG | 10 | 0.071108583 |
| CCAA/TTGG | 9 | 0.063997725 |
| GTCA/TGAC | 9 | 0.063997725 |
| AAGC/GCTT | 8 | 0.056886866 |
| AATC/GATT | 8 | 0.056886866 |
| CATC/GATG | 8 | 0.056886866 |
| CTTT/AAAG | 7 | 0.049776008 |
| CATA/TATG | 7 | 0.049776008 |
| CGAC/GTCG | 7 | 0.049776008 |
| AATT/AATT | 7 | 0.049776008 |
| ATTA/TAAT | 7 | 0.049776008 |
| TCAG/CTGA | 6 | 0.04266515 |
| TCCA/TGGA | 6 | 0.04266515 |
| TGAA/TTCA | 6 | 0.04266515 |
| ACCG/CGGT | 5 | 0.035554291 |
| GGAG/CTCC | 5 | 0.035554291 |
| TCGG/CCGA | 5 | 0.035554291 |
| TATGC/GCATA | 5 | 0.035554291 |
| GAAA/TTTC | 5 | 0.035554291 |
| TTAA/TTAA | 5 | 0.035554291 |
| CTGTT/AACAG | 5 | 0.035554291 |
| TGTTC/GAACA | 5 | 0.035554291 |
| AGAA/TTCT | 4 | 0.028443433 |
| GATC/GATC | 4 | 0.028443433 |
| AGTG/CACT | 4 | 0.028443433 |
| AGAAC/GTTCT | 4 | 0.028443433 |
| CCTC/GAGG | 4 | 0.028443433 |
| ATACA/TGTAT | 4 | 0.028443433 |
| ATTTG/CAAAT | 4 | 0.028443433 |
| CAGG/CCTG | 3 | 0.021332575 |
| ACCT/AGGT | 3 | 0.021332575 |
| TCGT/ACGA | 3 | 0.021332575 |
| GAGTG/CACTC | 3 | 0.021332575 |
| GAAG/CTTC | 3 | 0.021332575 |
| ACAAT/ATTGT | 3 | 0.021332575 |
| ACGG/CCGT | 3 | 0.021332575 |
| GTATT/AATAC | 3 | 0.021332575 |
| TTCG/CGAA | 3 | 0.021332575 |
| CATT/AATG | 3 | 0.021332575 |
| CTGC/GCAG | 3 | 0.021332575 |
| CCAT/ATGG | 3 | 0.021332575 |
| TCCC/GGGA | 3 | 0.021332575 |
| GAGT/ACTC | 3 | 0.021332575 |
| TTTTG/CAAAA | 2 | 0.014221717 |
| TCAC/GTGA | 2 | 0.014221717 |
| ACTTA/TAAGT | 2 | 0.014221717 |
| TTGTA/TACAA | 2 | 0.014221717 |
| TTAC/GTAA | 2 | 0.014221717 |
| CACACG/CGTGTG | 2 | 0.014221717 |
| TGGTT/AACCA | 2 | 0.014221717 |
| GTGTG/CACAC | 2 | 0.014221717 |
| TTAG/CTAA | 2 | 0.014221717 |
| TAACA/TGTTA | 2 | 0.014221717 |
| TGCAG/CTGCA | 2 | 0.014221717 |
| TACC/GGTA | 2 | 0.014221717 |
| TTTGT/ACAAA | 2 | 0.014221717 |
| CTGG/CCAG | 2 | 0.014221717 |
| ATAC/GTAT | 2 | 0.014221717 |
| TTGTG/CACAA | 2 | 0.014221717 |
| CCTCC/GGAGG | 2 | 0.014221717 |
| GAGC/GCTC | 2 | 0.014221717 |
| GTTTG/CAAAC | 2 | 0.014221717 |
| GACTA/TAGTC | 2 | 0.014221717 |
| TCCT/AGGA | 2 | 0.014221717 |
| ACAT/ATGT | 2 | 0.014221717 |
| TTCTG/CAGAA | 2 | 0.014221717 |
| AAACA/TGTTT | 2 | 0.014221717 |
| TTGGT/ACCAA | 2 | 0.014221717 |
| ACTA/TAGT | 2 | 0.014221717 |
| ACTG/CAGT | 1 | 0.007110858 |
| TCTTG/CAAGA | 1 | 0.007110858 |
| CACGA/TCGTG | 1 | 0.007110858 |
| GTTTA/TAAAC | 1 | 0.007110858 |
| CTTG/CAAG | 1 | 0.007110858 |
| CCG/CGG | 1 | 0.007110858 |
| AACAT/ATGTT | 1 | 0.007110858 |
| GTACG/CGTAC | 1 | 0.007110858 |
| ATCG/CGAT | 1 | 0.007110858 |
| GCCAC/GTGGC | 1 | 0.007110858 |
| TACG/CGTA | 1 | 0.007110858 |
| GGCA/TGCC | 1 | 0.007110858 |
| GTAAA/TTTAC | 1 | 0.007110858 |
| AACAC/GTGTT | 1 | 0.007110858 |
| ATGAC/GTCAT | 1 | 0.007110858 |
| TAGA/TCTA | 1 | 0.007110858 |
| ATGGT/ACCAT | 1 | 0.007110858 |
| TTCTA/TAGAA | 1 | 0.007110858 |
| TATC/GATA | 1 | 0.007110858 |
| TCTGT/ACAGA | 1 | 0.007110858 |
| AGTGGCCTT/AAGGCCACT | 1 | 0.007110858 |
| ATTC/GAAT | 1 | 0.007110858 |
| TGGC/GCCA | 1 | 0.007110858 |
| GTGTGC/GCACAC | 1 | 0.007110858 |
| TCGC/GCGA | 1 | 0.007110858 |
| GGTGT/ACACC | 1 | 0.007110858 |
| CAATC/GATTG | 1 | 0.007110858 |
| ATCC/GGAT | 1 | 0.007110858 |
| TTATC/GATAA | 1 | 0.007110858 |
| TGCT/AGCA | 1 | 0.007110858 |
| TGCCC/GGGCA | 1 | 0.007110858 |
| CAGAT/ATCTG | 1 | 0.007110858 |
| CATAA/TTATG | 1 | 0.007110858 |
| AATTC/GAATT | 1 | 0.007110858 |
| GTAAT/ATTAC | 1 | 0.007110858 |
| CTAC/GTAG | 1 | 0.007110858 |
| AACT/AGTT | 1 | 0.007110858 |
| TTCGT/ACGAA | 1 | 0.007110858 |
| AACAA/TTGTT | 1 | 0.007110858 |
| GGCGT/ACGCC | 1 | 0.007110858 |
| AGCAC/GTGCT | 1 | 0.007110858 |
| TGTGC/GCACA | 1 | 0.007110858 |
| GTGAA/TTCAC | 1 | 0.007110858 |
| TGTAG/CTACA | 1 | 0.007110858 |
| TATCG/CGATA | 1 | 0.007110858 |
| AAGA/TCTT | 1 | 0.007110858 |
| ATATTCATGC/GCATGAATAT | 1 | 0.007110858 |
| AGTA/TACT | 1 | 0.007110858 |
| CGAG/CTCG | 1 | 0.007110858 |
| GCGTGT/ACACGC | 1 | 0.007110858 |
| ACATC/GATGT | 1 | 0.007110858 |
| ACAGT/ACTGT | 1 | 0.007110858 |
| Total above | Total above | Total above |
| 187 | 14063 | 100 |

| Supplementary Table 4: SSR loci length and respective loci number recognized by GMATA | | |
| --- | --- | --- |
| **SSR loci length** | **Total loci** | **Percentage** |
| 10 | 5249 | 37.32489511 |
| 15 | 1884 | 13.396857 |
| 12 | 1289 | 9.165896324 |
| 24 | 906 | 6.442437602 |
| 18 | 863 | 6.136670696 |
| 20 | 690 | 4.906492214 |
| 14 | 620 | 4.408732134 |
| 21 | 402 | 2.858565029 |
| 16 | 384 | 2.73056958 |
| 22 | 143 | 1.016852734 |
| 28 | 120 | 0.853302994 |
| 30 | 108 | 0.767972694 |
| 48 | 91 | 0.647088104 |
| 32 | 90 | 0.639977245 |
| 36 | 84 | 0.597312096 |
| 40 | 77 | 0.547536088 |
| 60 | 66 | 0.469316647 |
| 25 | 65 | 0.462205788 |
| 54 | 64 | 0.45509493 |
| 42 | 60 | 0.426651497 |
| 44 | 57 | 0.405318922 |
| 56 | 51 | 0.362653772 |
| 33 | 50 | 0.355542914 |
| 52 | 49 | 0.348432056 |
| 38 | 47 | 0.334210339 |
| 26 | 47 | 0.334210339 |
| 57 | 44 | 0.312877764 |
| 27 | 44 | 0.312877764 |
| 39 | 37 | 0.263101756 |
| 34 | 37 | 0.263101756 |
| 51 | 36 | 0.255990898 |
| 64 | 35 | 0.24888004 |
| 63 | 29 | 0.20621489 |
| 46 | 27 | 0.191993174 |
| 72 | 24 | 0.170660599 |
| 50 | 23 | 0.16354974 |
| 45 | 20 | 0.142217166 |
| 66 | 20 | 0.142217166 |
| 35 | 19 | 0.135106307 |
| 58 | 18 | 0.127995449 |
| 80 | 14 | 0.099552016 |
| 68 | 11 | 0.078219441 |
| 69 | 10 | 0.071108583 |
| 78 | 10 | 0.071108583 |
| 76 | 9 | 0.063997725 |
| 62 | 8 | 0.056886866 |
| 75 | 5 | 0.035554291 |
| 84 | 5 | 0.035554291 |
| 55 | 4 | 0.028443433 |
| 70 | 3 | 0.021332575 |
| 100 | 3 | 0.021332575 |
| 93 | 2 | 0.014221717 |
| 102 | 2 | 0.014221717 |
| 90 | 2 | 0.014221717 |
| 110 | 1 | 0.007110858 |
| 104 | 1 | 0.007110858 |
| 65 | 1 | 0.007110858 |
| 96 | 1 | 0.007110858 |
| 92 | 1 | 0.007110858 |
| 87 | 1 | 0.007110858 |
| Total above | Total above | Total above |
| 60 | 14063 | 100 |
